# Giant cell tumor of bone at distal radius suffered more soft tissue recurrence and ultrasonography is effective to detect the soft tissue recurrence

**DOI:** 10.1007/s12672-024-00918-0

**Published:** 2024-04-04

**Authors:** Lukuan Cui, Yang Sun, Tao Jin, Daoyang Fan, Weifeng Liu

**Affiliations:** 1 Department of Bone and Soft Tissue Oncology, Cangzhou Hospital of Integrated TCM-WM·Hebei, No. 5, Xianghai Road, Cangzhou, 061000 Hebei People’s Republic of China; 2 Hebei Key Laboratory of Integrated Traditional and Western Medicine in Osteoarthrosis Research (Preparing), No. 5, Xianghai Road, Cangzhou, 061000 Hebei People’s Republic of China; 3 Department of Orthopedic Oncology, Beijing Jishuitan Hospital Affiliated to Capital Medical University, No. 31, Xinjiekou East Steet, Xicheng District, Beijing, 100035 People’s Republic of China

**Keywords:** Giant cell tumor of bone, Soft tissue, Recurrence, Ultrasonography, Risk factor

## Abstract

**Background:**

Soft tissue recurrence of giant cell tumor of bone (GCTB) is rare. This study aims to provide its prevalence, recurrent locations, risk factors, effective detection methods and a modified classification for this recurrence.

**Methods:**

Patients with soft tissue recurrence after primary surgery for GCTB were screened from January 2003 to December 2022. General data, recurrence frequency, types according to an original classification (type-I: peripheral ossification; type-II: central ossification; type-III: without ossification), a modified classification with more detailed subtypes (type I-1: ≤ 1/2 peripheral ossification; type I-2: ≥ 1/2 peripheral ossification; type II-1: ≤ 1/2 central ossification; type II-2: ≥ 1/2 central ossification; type III: without ossification), locations, detection methods such as ultrasonography, X-ray, CT or MRI, Musculoskeletal Tumor Society (MSTS) scores were recorded. Multivariate regression analysis was conducted to identify risk factors for recurrence frequency.

**Results:**

A total of 558 recurrent cases were identified from 2009 patients with GCTB. Among them, 32 were soft tissue recurrence. The total recurrence rate was 27.78% (558/2009). Soft tissue recurrence rate was 5.73% among 558 recurrent cases, and 1.59% among 2009 GCTB patients, respectively. After excluding one patient lost to follow-up, 10 males and 21 females with the mean age of 28.52 ± 9.93 (16–57) years were included. The definitive diagnosis of all recurrences was confirmed by postoperative pathology. The interval from primary surgery to the first recurrence was 23.23 ± 26.12 (2–27) months. Eight recurrences occurred from primary GCTB located at distal radius, followed by distal femur (6 cases). Recurrence occurred twice in 12 patients and 3 times in 7 patients. Twenty-seven recurrences were firstly detected by ultrasonography, followed by CT or X-ray (10 cases in each). Types at the first recurrence were 5 cases in type-I, 8 in type-II and 18 in type-III. According to the modified classification, 3 patients in type I-1, 2 in type I-2, 1 in type II-1, 7 in type II-2, and 18 in type III. The mean MSTS score was 26.62 ± 4.21 (14—30). Neither Campanacci grade nor recurrence type, modified classification and other characters, were identified as risk factors.

**Conclusions:**

Soft tissue recurrence of GCTB may recur for more than once and distal radius was the most common location of primary GCTB that would suffer a soft tissue recurrence. Ultrasonography was a useful method to detect the recurrence. Since no risk factors were discovered, a careful follow-up with ultrasonography was recommended.

## Background

Giant cell tumor of bone (GCTB) is a benign lesion which generally affects meta-epiphyseal region of long bones in adults aged from 20 to 40 years. Since it accounts for approximately 5% of all tumors primarily occurred in bone, [1] GCTB is not a rare neoplasm in clinic. Surgical treatment for GCTB includes resection and curettage combined with adjunct procedures like burring or bone cement application. Although various adjunct procedures have been developed, surgical treatment was still a challenge for clinicians owing to the locally aggressive behavior and high recurrence rate of GCTB [2]. GCTB recurrence would sometimes undergo malignant transformation or metastasis to lung or other locations, and subsequently leading to adverse sequelae like death or amputation [3].

Local recurrence of GCTB in bone, its inducing factors and treatment methods have been extensively studied and widely reported [4, 5]. However, due to the low incidence rate, soft tissue recurrence after operation of GCTB has not received sufficient attention. Research in this area was less. But this phenomenon should not be ignored as it also has significant implications for patients [6].

Recently, Xu and his colleagues [7] reported 6 patients with soft tissue recurrence of GCTB and presented a classification system for this recurrence. In their study, recurrent lesions of soft tissue were classified into 3 types according to radiological features: type I was defined as a soft tissue lesion with peripheral ossification; type II as central ossification in recurrence tissue; and type III as pure soft tissue lesion with no ossification. Although it was the first classification system specially used for soft tissue recurrence of GCTB, we found it was not detailed enough for clinical practice. For example, patients with ossification less or more than half of the lesion rim were not distinguished. The same situation could also be found in patients with central ossification.

In this study, we retrospectively analyzed the soft tissue recurrence after primary surgical treatment of GCTB. The aim of this study was to evaluate the recurrent prevalence, characteristics, risk factors and suitable detection methods. At the same time, we testified the classification mentioned above, as well as a modified classification based on that system. Hypotheses of this study were that some factors may contribute to multiple times of soft tissue recurrence of GCTB, and the modified classification system could better reflect prognosis of recurrence frequency.

## Material and methods

From January 2003 to December 2022, patients with GCTB admitted to our center were screened and patients with soft tissue recurrence were selected. All soft tissue recurrence were confirmed by pathological examination after excision. Demographic data, primary and recurrent locations, Campanacci grades before primary surgical treatment, types of soft tissue recurrence, intervals of soft tissue recurrence, follow-up intervals, detection methods for recurrent soft tissue lesions, number of soft tissue recurrence times and the Musculoskeletal Tumor Society (MSTS) scores were recorded for further analysis.

**Fig. 1:**
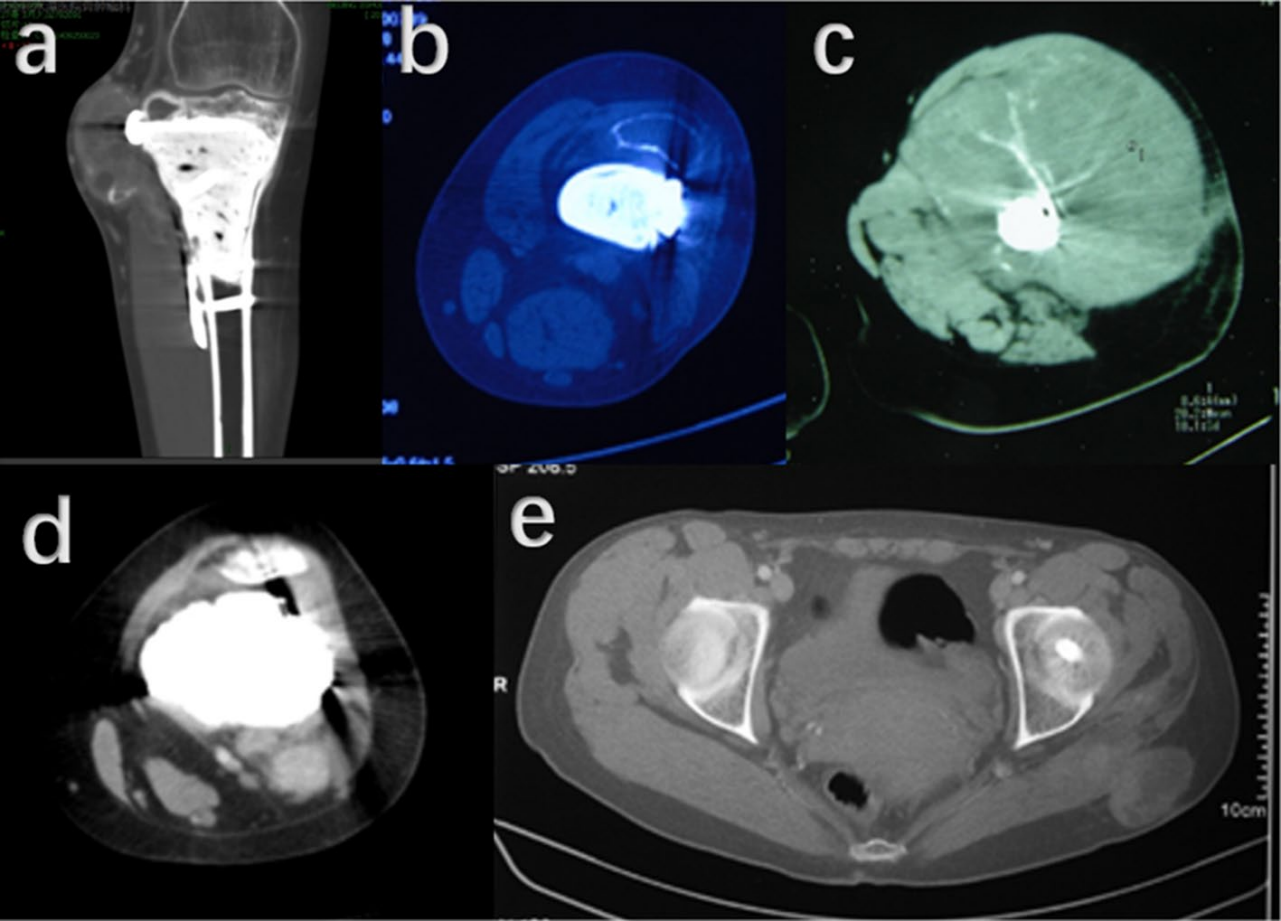
Types of modified classification system. **a** Type I-1, a soft tissue recurrence lesion with ossified peripheral rim less than 1/2 circle. **b** Type I-2, an eggshell like ossification rim surrounding the recurrent soft mass. **c** Type II-1, central ossification in soft tissue but less than 1/2 of the whole lesion. **d** TypeII-2, ossification occupied the whole area of recurrent soft mass. **e** Type III, a pure soft tissue mass without ossification

Age of the patients was recorded as years at the first time of surgery. Since some patients received primary surgery for GCTB in other institutions, to eliminate bias, the methods of primary operation were not evaluated for contribution to recurrence. Because the method of classifying soft tissue recurrence into 3 types may not be detailed enough to evaluate contribution of soft tissue lesion severity for re-recurrence, we further divided these 3 types into 5 levels: type I-1 as soft tissue recurrence lesion with peripheral ossification but less than half of the peripheral rim, while type I-2 as more than 1/2 of peripheral ossification, including the typical “egg-shell” like ossification rim; type II-1 as a central ossification located in soft tissue lesion but less than half of the mass, and type II-2 as ossification occupied from half to the whole lesion; type III was the same as previous presenters: no ossification could be observed at all in recurrent soft tissue mass. (Fig. 1 and Table 1) This new classification was also recorded to testify risk factors for soft tissue recurrence times.

**Table 1:** Modified classification method for soft tissue recurrence of GCTB

Type	Description
Type I-1	Peripheral ossification, less than 1/2 of the mass rim
Type I-2	Peripheral ossification, 1/2 of the rim to an “egg-shell” like ossification rim
Type II-1	Central ossification, less than 1/2 of the soft tissue lesion
Type II-2	Central ossification, 1/2 to the whole area of soft tissue lesion
Type III	No ossification within the soft tissue recurrence mass

This study was approved by Ethics Committee of our institution and all procedures were performed in accordance with the ethical standards as laid down in the 1964 Declaration of Helsinki and its later amendments or comparable ethical standers. All patients included or their kinsfolks were informed and agreed to participate in this study for the retrospective analysis of their outcomes after surgery.

SPSS 22.0 software (IBM, Armonk, NY, USA) was used to calculate mean and standard deviation for continuous variables such as age and follow-up interval, and rate or ratio for recurrence times, gender and other categorical variables. Multivariate regression analysis was applied to detect risk factors for number of soft tissue recurrence times and a significant level of *P* value < 0.05 was set.

GCTB: giant cell tumor of bone.

## Results

After screening database of our bone and soft tissue tumor center, a number of 2009 patients with GCTB were treated in our institution during this study interval. Among them, 558 patients suffered recurrent GCTB of bone or soft tissue. The total recurrence rate of GCTB including recurrence both in bone and soft tissue was 27.78% (558/2009). After excluding one patient lost to follow-up, 31 patients with soft tissue recurrence were included for further analysis. The rate of soft tissue recurrence in all GCTB patients was 1.59% (32/2009), and 5.73% (32/558) in recurrent patients, respectively. General characteristics of these 31 patients were presented in Table 2. Surgical procedures for the primary GCTB were presented in Table 3. Characteristics of the lesion including primary Campanacci grades, number of soft tissue recurrence times, types of recurrence lesion according to Xu et al., [7] and new types according to our modified classification were showed in Table 4. From the tables, we could see that patients aged from 20 to 40 years accounted for 67.64% (21/31), distal radius and distal femur suffered more soft tissue recurrence (with 8 and 6 cases, respectively) than other single locations. Locations around the knee (distal femur + proximal tibia + proximal fibula, 13 cases) and wrist (distal radius + distal ulna, 12 cases) had more opportunity to suffer soft tissue recurrence. Campanacci grade III was the most common grade in primary GCTB. The recurrence occurred once and twice each in 12 patients, and three times in 7 patients. Nineteen patients (61.29%) underwent a soft tissue recurrence more than once. These 57 recurrent lesions in 31 patients were firstly detected by palpation in 4 (7.02%), ultrasonography in 27 (47.37%), CT in 10 (17.54%), ECT in 1 (1.75%), X-ray in 10 (17.54%), MRI in 2 (3.51%). The method by which the lesions were first detected was not documented in the remaining 3 cases (5.26%).

**Table 2:** General characteristics of 31 patients with soft tissue recurrence of GCTB

Characteristics	n (%)
Gender	
Male	10 (32.26%)
Female	21 (67.74%)
Age (years)	
≤ 20	7 (22.58%)
20–30	11 (35.38%)
30–40	10 (32.26%)
40–50	2 (6.45%)
> 50	1 (3.23%)
Location of primary tumor	
Proximal femur	3 (9.68%)
Distal femur	6 (19.35%)
Proximal tibia	3 (9.68%)
Proximal fibula	4 (12.90%)
Distal radius	8 (25.81%)
Distal ulna	4 (12.90%)
First metacarpal	1 (3.23%)
First metatarsal	1 (3.23%)
Patella	1 (3.23%)
Follow-up interval (months)	115.48 ± 52.56 (15–229)

GCTB: giant cell tumor of bone

**Table 3:** Surgical procedures for the primary GCTB

Location of primary tumor	Surgical procedure	Number of patients (n = 31)
Proximal femur	Curettage + DHS + PMMA	1
	Curettage + PMMA	1
	Resection + arthroplasty	1
Distal femur	Curettage + bone graft + plate	2
	Curettage + bone graft + PMMA + plate	2
	Curettage + PMMA + plate	1
	Curettage + PMMA	1
Proximal tibia	Curettage + PMMA + plate	1
	Curettage + bong graft	1
	Curettage + bone + PMMA + plate	1
Proximal fibular	Resection	4
Distal radius	Curettage + bone graft + external fixator	1
	Resection + fibular graft + plate	1
	Curettage + PMMA	1
	Curettage + bone graft	1
	Resection + autologous iliac graft + plate	3
	Curettage + artificial bone + PMMA	1
Distal ulna	Resection	4
First metacarpal	Curettage + bone graft + plate	1
First metatarsal	Curettage + bone graft + plate	1
Patella	Curettage + bone graft	1

DHS: dynamic hip screw; PMMA: polymethylmethacrylate bone cement

**Table 4:** Characteristics of primary GCTB and soft tissue recurrence

Characteristics	n (%)
Campanacci grade	
I	2 (6.45%)
II	9 (29.03%)
III	20 (64.52%)
Number of soft tissue recurrence time	
Once	12 (38.71%)
Twice	12 (38.71%)
Thrice	7 (22.58%)
Type of soft tissue recurrence	
I	5 (16.13%)
II	8 (25.81%)
III	18 (58.06%)
New classification type of soft tissue recurrence	
I-1	3 (9.68%)
I-2	2 (6.45%)
II-1	1 (3.23%)
II-2	7 (22.58%)
III	18 (58.06%)

GCTB: giant cell tumor of bone

Interval between primary surgery for GCTB and the first time of soft tissue recurrence was 23.23 ± 26.12 (2–127) months, while interval between re-operation for the first recurrence and the second time of soft tissue recurrence (12 patients) was 20.89 ± 18.09 (4–72) months. For the 7 patients underwent 3 times of recurrence, latent period of the first recurrence was 27.00 ± 27.00 (2–73) months, interval period of the second recurrence was 25.57 ± 21.69 (10–72) months, and interval of the third recurrence was 16.71 ± 12.46 (3–31) months. The latent period of soft tissue recurrence shortened gradually from the first to the third recurrence.

To investigate which factor would affect soft tissue recurrence, multivariate regression analysis was applied to assess contributions of gender, age group, Campanacci grades, recurrence interval of the first time, as well as the originate and new classification types of soft tissue recurrence. However, final results showed none factor could independently impact number of soft tissue recurrence time after surgery for GCTB (Table 5).

**Table 5:** Regression analysis results of factors for soft tissue recurrence time

Factors	β	T	*P*
Gender	0.378	1.928	0.066
Campanacci grade	− 0.006	− 0.030	0.976
Age group	0.044	0.205	0.839
Interval of the first recurrence	0.188	0.995	0.330
Type of recurrence lesion	0.123	0.210	0.835
New type of recurrence lesion	0.388	0.650	0.522

GCTB: giant cell tumor of bone

**Fig. 2:**
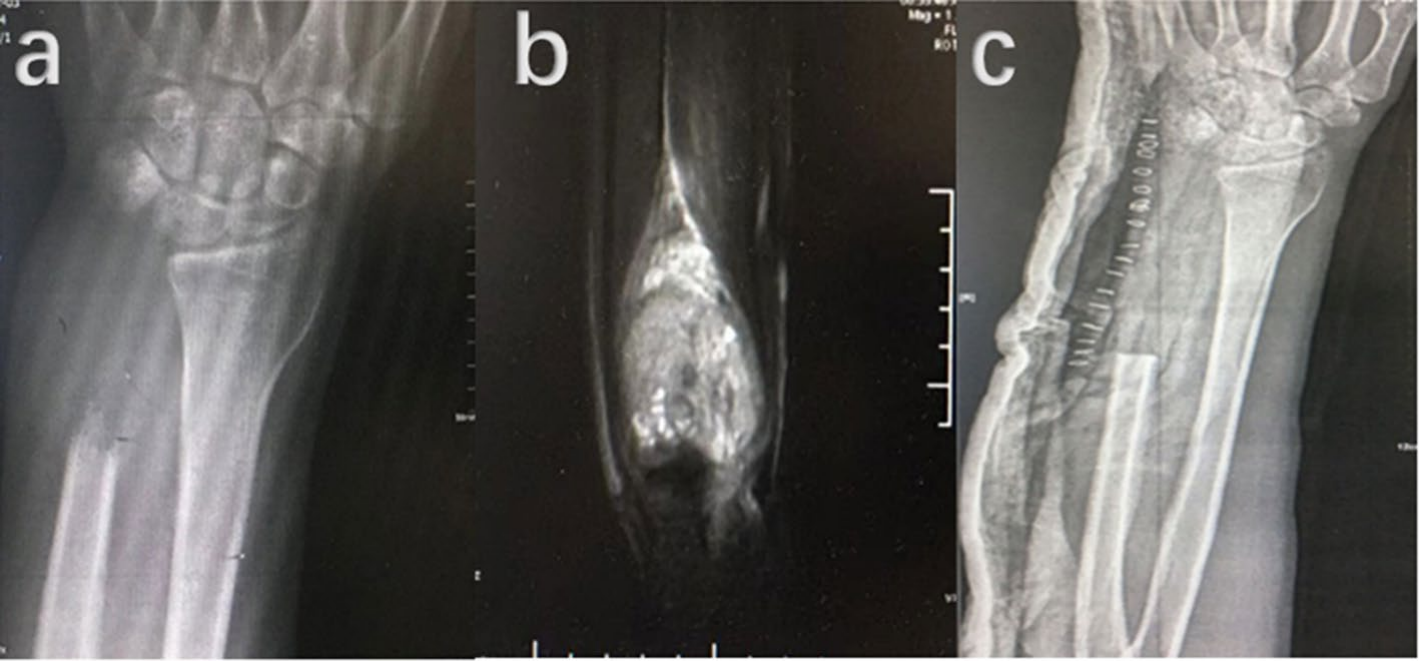
Radiographic images of a 24-year-old male patient with GCTB located in distal ulna at the time of primary surgery. **a** Plain radiography before operation showed osteolytic destruction at left distal ulna. **b** MRI revealed intermediate to high intensity signal in the lesion. **c** Post-operative plain radiography after distal ulna has been resected

**Fig. 3:**
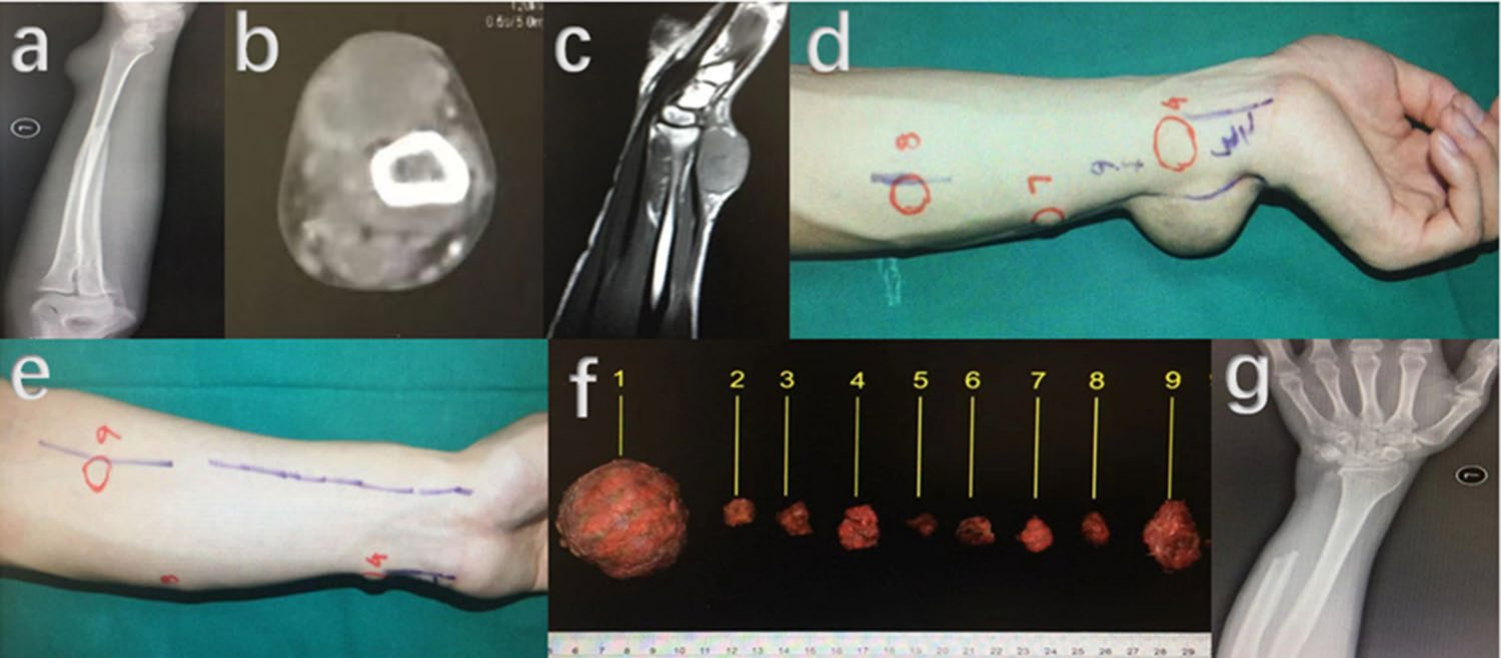
Images at the first time of soft tissue recurrence of the 24- year-old male patient with GCTB located in distal ulna after an interval of 5 months. **a** Plain radiography showed a soft tissue mass in dorsal forearm. **b** A solid mass could be seen on CT image. **c** Intermediate signal was presented on T-1 weighted MRI. **d** and **e** Locations of multiple soft tissue recurrence masses in forearm.** f**: Resected specimens of the nine recurrent masses. **g**: Post-operative X-ray radiography

**Fig. 4:**
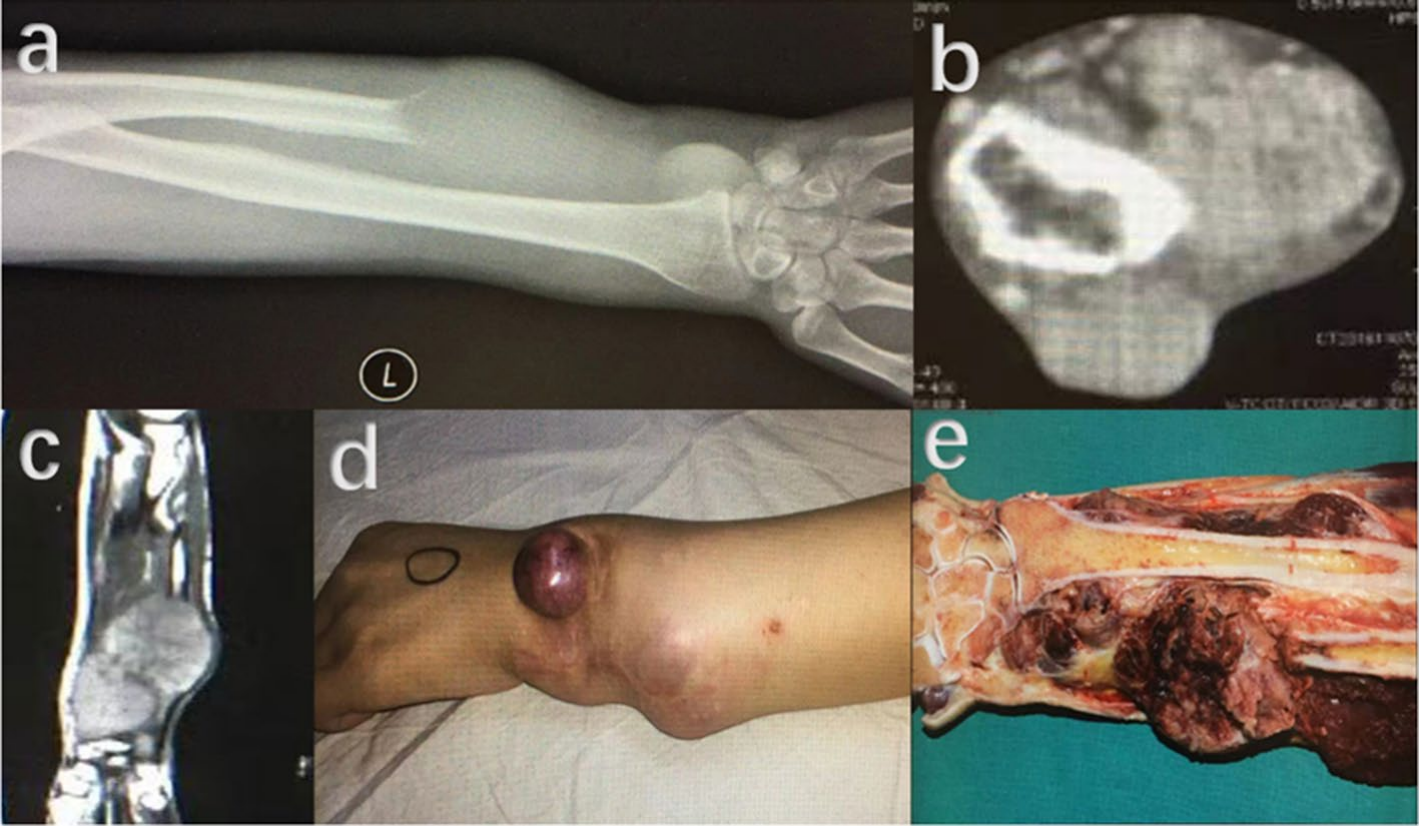
Nine months later, soft tissue lesion reoccurred after the second surgery of the 24- year-old male patient with GCTB located in distal ulna. **a**–**c** X-ray, CT and MRI presentations of re-recurrent soft tissue mass in the same patient. **d** and **e** Image before the third operation and specimen after the operation, a protruding mass in dorsal forearm and a mass occupied the space left by ulna resection could be observed

**Fig. 5:**
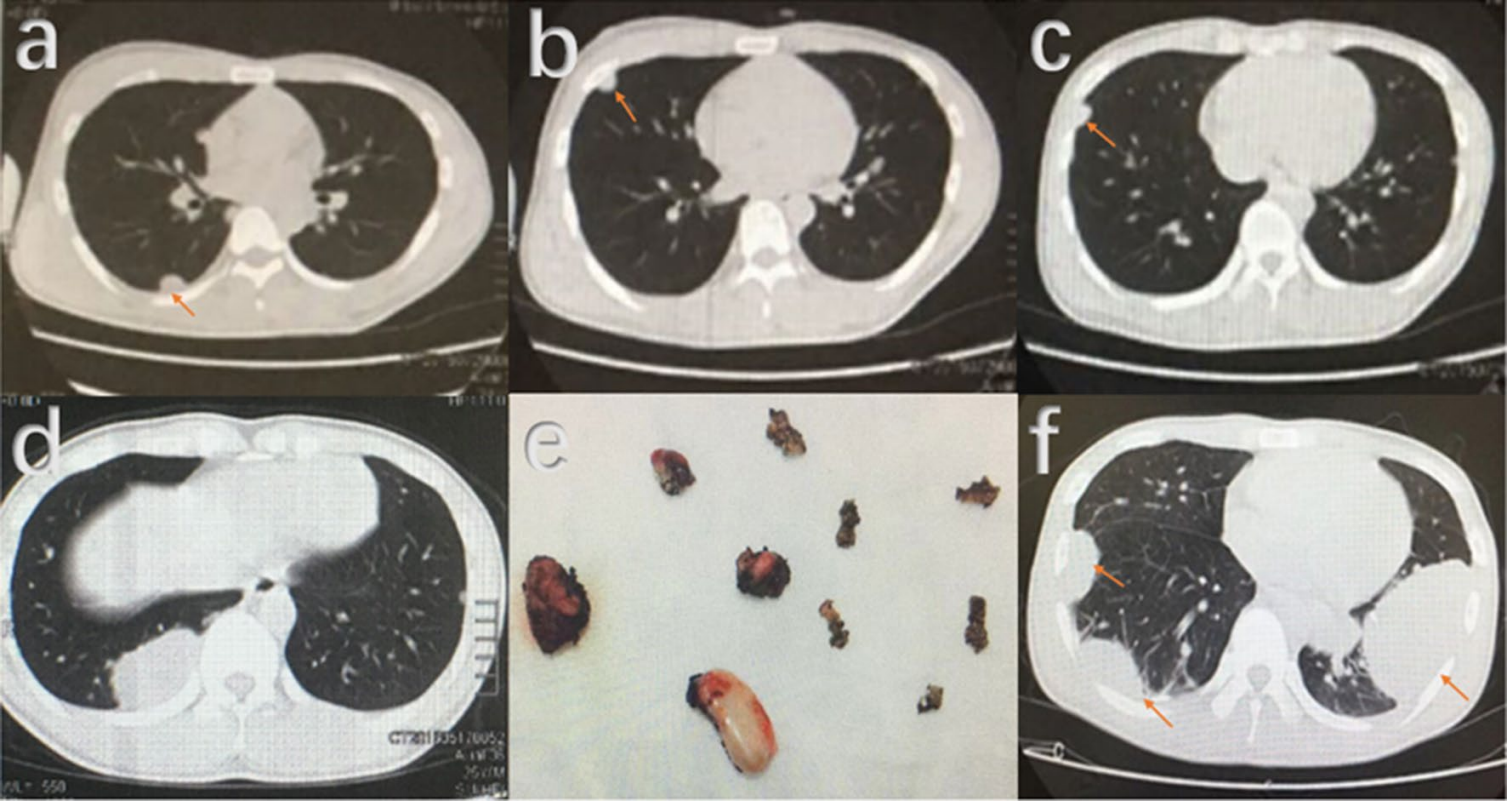
Progression of lung metastasis of the 24- year-old male patient with GCTB located in distal ulna. **a**–**c** Pulmonary nodules (arrows) were found with a follow-up of 5 months after primary surgery in distal ulna. **d** Pulmonary metastasis progresses 10 months later. **e** Metastasis lesions in lung were resected 14 months after the primary surgery for ulna GCTB. **f** Unfortunately, pulmonary metastasis reoccurred and progressed (arrows) during a follow-up period of 12 months after operation on the lung. At last, the patients died with pulmonary metastasis

After excluding a death caused by pulmonary metastasis from GCTB (Figs. 2, 3, 4, 5) and an amputation in the upper thigh, there were 29 patients left to be assessed with MSTS scoring system. The mean follow-up period of these patients was 115.48 ± 52.56 (15–229) months, and the mean MSTS score was 26.62 ± 4.21 (14–30). In patients with three times recurrence, an average MSTS score of 25.43 ± 4.96 (16–30) was received. Four of them had pulmonary nodules in chest CT, although lung metastasis was not confirmed by pathology.

## Discussion

To the best of our knowledge, in this study, we reported the largest number of patients with pure soft tissue recurrence of GCTB in a single center. Meanwhile, we testified predictive value of recurrence types and a modified classification system for this soft tissue recurrence. Although our results showed that types of recurrence may not affect number of soft tissue recurrence times, we still believe that this new classification would be helpful for surgeons to deeply assess and chose a better examination tool to detect these lesions. Our results also revealed the effectiveness of ultrasonography in detection of soft tissue recurrence of GCTB.

An accurate soft tissue recurrence rate of GCTB has not been revealed. GCTB has a high local recurrence rate [8], distant metastasis and malignant transformation could also be observed in patients with GCTB [9]. Previous studies have recognized the phenomenon of soft tissue recurrence after primary operation for GCTB, but their samples were small or mixed with local recurrence in bone to analyze. Thus, an accurate incidence rate and risk factors could not be evaluated exactly. Previously, 17 cases with soft tissue recurrence were reported by Cooper et al. [6] among 1100 patients with GCTB, the rate of soft tissue recurrence was 1.5%, which was similar with our results. A rate of 3.1% (4 patients among 129 GCTB cases) was reported by Park et al., [10] and 2.1% (6 soft tissue recurrent cases in 291 patients with GCTB) by Xu et al. 7] Another 3 cases were described by Ehara et al. [11] and 4 cases by Lee et al. [12] A concern about extending these incidence rate to other centers is that these cases were mainly detected by radiography imaging when ossification or mineralization could be found on X-ray or CT. While some candidates without ossification may have been missed.

Many methods could be used to detect recurrence of soft tissue in GCTB. If recurrent soft tissue mass located at a body region with less muscle or fat tissue, like the forearm, it could be detected by palpation and an ultrasound examination would be enough. Detection of this lesion by plain radiograph was difficult, even in some cases with peripheral mineralization [13]. In this situation, CT or MRI may be a preferred method. Inhomogeneous low to intermediate or high signal intensity could be observed in recurrent soft tissue lesions on different weight or fat-suppressed MRI series [7, 10]. But MRI was less sensible than CT in observing ossification. In our results, 27 of all 57 recurrent lesions were firstly detected by ultrasonography, followed by CT and X-ray. This may be explained by making full use of ultrasound examination during follow-up at our department. The results indicated the effectiveness of ultrasonography in distinguishing soft tissue recurrence of GCTB in clinical practice, when recurrent mass was not obvious in radiograph or other radiological exsminstions were postponed. Further research could focus on the ultrasound imaging characteristics of soft tissue recurrent lesions of GCTB, and compare the sensitivity of ultrasonography with X-ray or CT examination in these patients, so as to provide a simple, fast and economical examination method for clinicians to detect such recurrence as soon as possible.

The original classification for soft tissue recurrent lesions introduced be Xu et al. [7] was proposed by a combination of MRI and plain radiograph. During practical application, we found that in spite of its simplicity and practicability, this classification may not be detailed enough to distinguish some cases with no typical eggshell like peripheral rim or overall central ossification. Therefore, we modified the original classification into 5 types based on features of recurrent soft lesions on CT. In our system, the original type I and II were further divided into more detailed types in order to provide surgeons a better guidance. However, our results showed that neither the original nor our modified classification system could contribute to number of recurrence. On the other hand, size of soft tissue recurrence lesion was not taken into consideration in the original and our modified classification, this may also contribute to the poor predictive effect of these two classification systems. Therefore, further studies are needed to develop a more refined classification system with better practicability and accuracy.

Due to various sizes, locations and extending to other surrounding tissues such as nerves, there was no one operation method could be appropriate for all recurrent patients. Adjuvant techniques, like burring or cementation, combined with intralesional curettage could help to reduce recurrence rate of GCTB in bone [14]. But this may not be suitable for soft tissue recurrence, since some lesions have no eggshell like ossification rims. Marginal excision remains mainstay surgical treatment for soft tissue recurrence. In the study of Xu et al., [7] all six patients with recurrent mass in soft tissue had received a marginal resection and none of them suffered a re-recurrence again. En bloc resection technique was also applied to reduce recurrence of primary GCTB with soft tissue extension. Generally, en bloc resection was deemed as a more effective manner than curettage in reducing recurrence rate. Therefore, if local recurrence was observed in a patient following an en bloc resection, this GCTB could be indicated as an aggressive type and a careful follow-up was recommended [15].

**Fig. 6:**
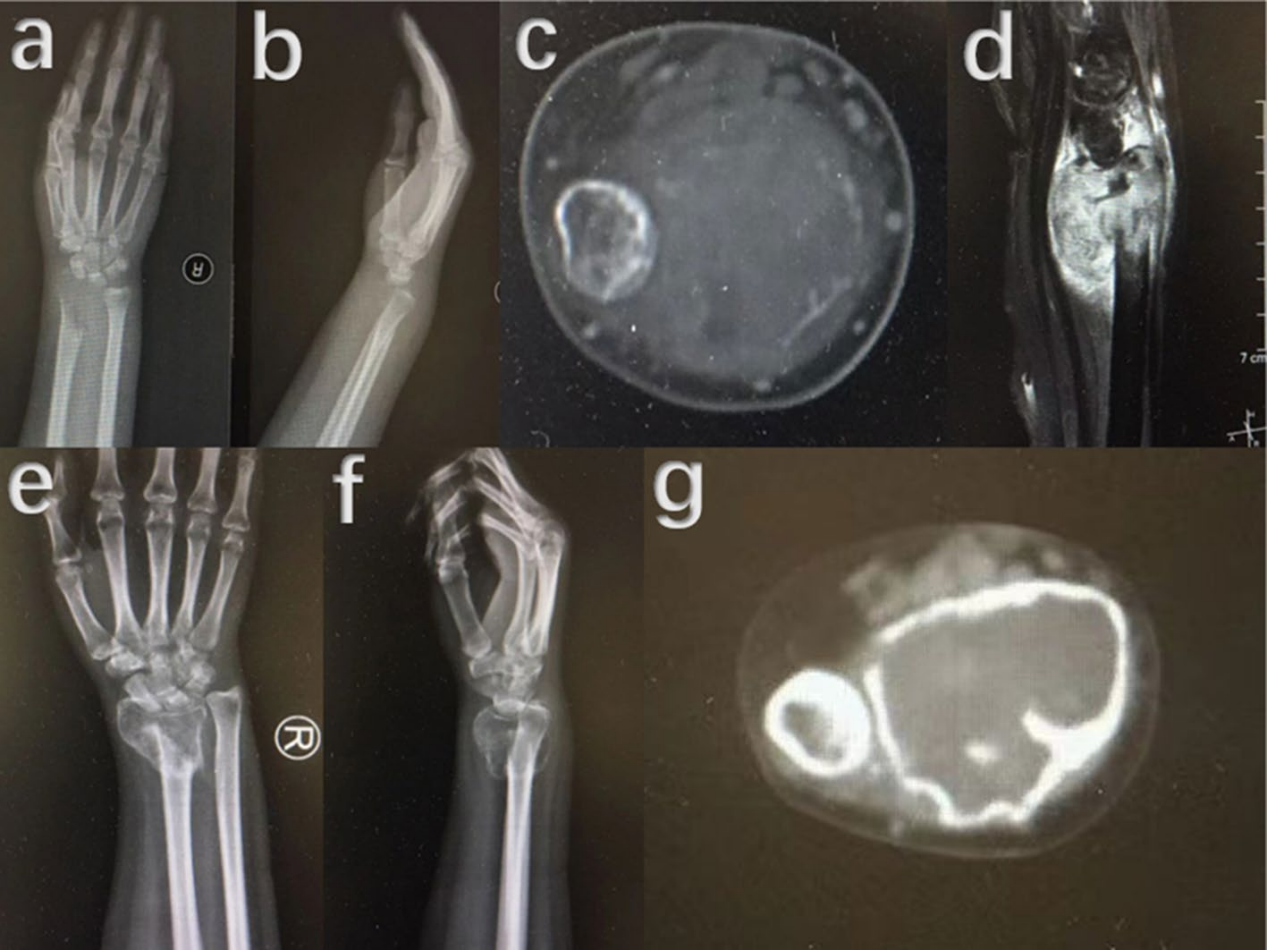
Preoperative images of another 22 years old female patient with GCTB located in distal radius. **a**–**d** Osteolytic destruction in right distal radius were revealed by X-ray (**a**, **b**), CT (**c**) and MRI (**d**). **e**–**g** Ossification could be observed on X-ray radiography (**e**, **f**) and CT (**g**) after application of denosumab for 2 months

**Fig. 7:**
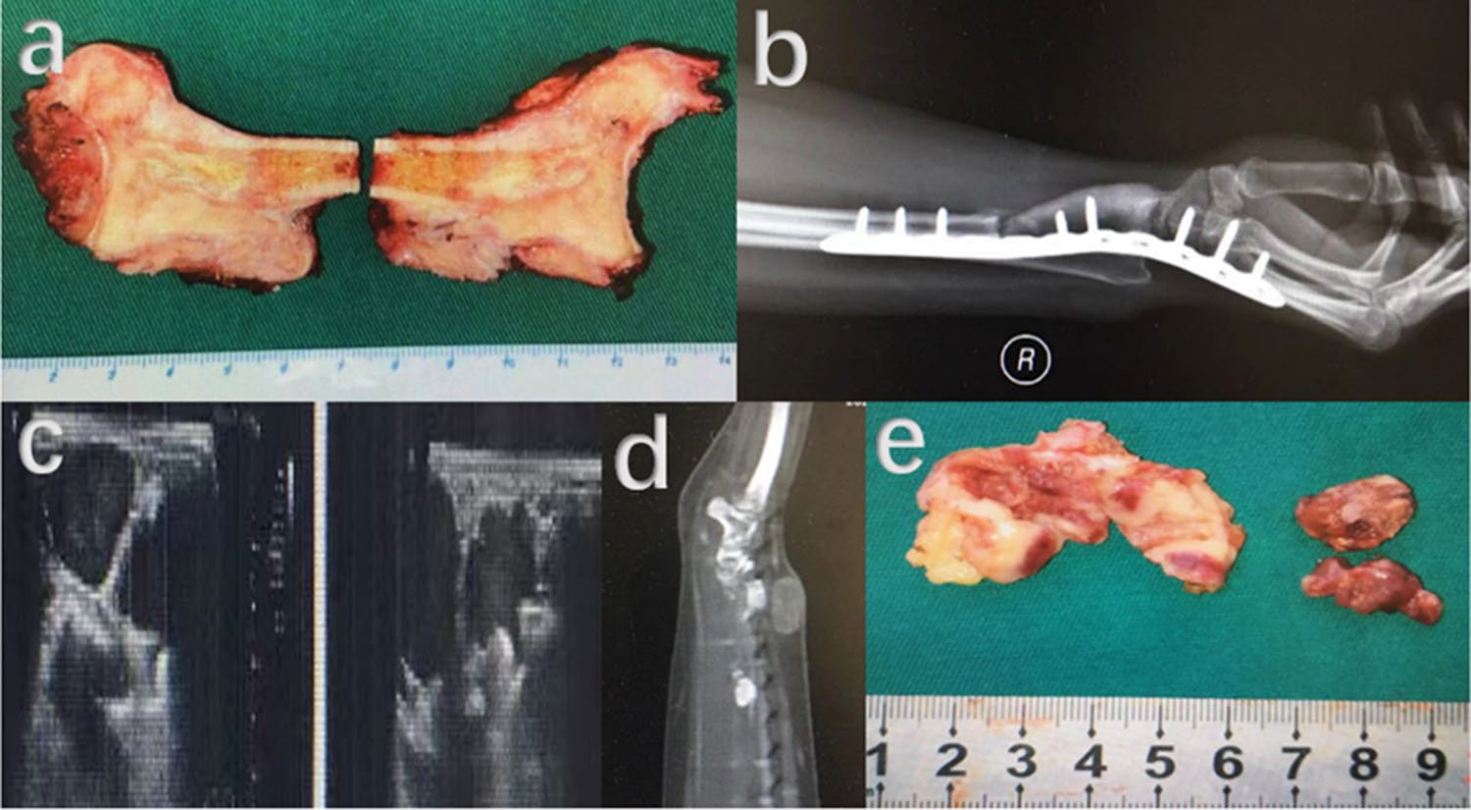
Although denosumab and widely resection had been applied, soft tissue recurrence still occurred after primary surgery. **a** Specimen of GCTB in distal radius with wide resection. **b** Post-operative plain radiography showing a wrist fusion with autogenous iliac bone graft and internal fixation were performed. **c** Hypoechoic mass could be detected around the original operation site by ultrasound. **d** Recurrent lesion could be obviously observed on CT. **e**: Specimens of excised soft tissue recurrence lesions

**Fig. 8:**
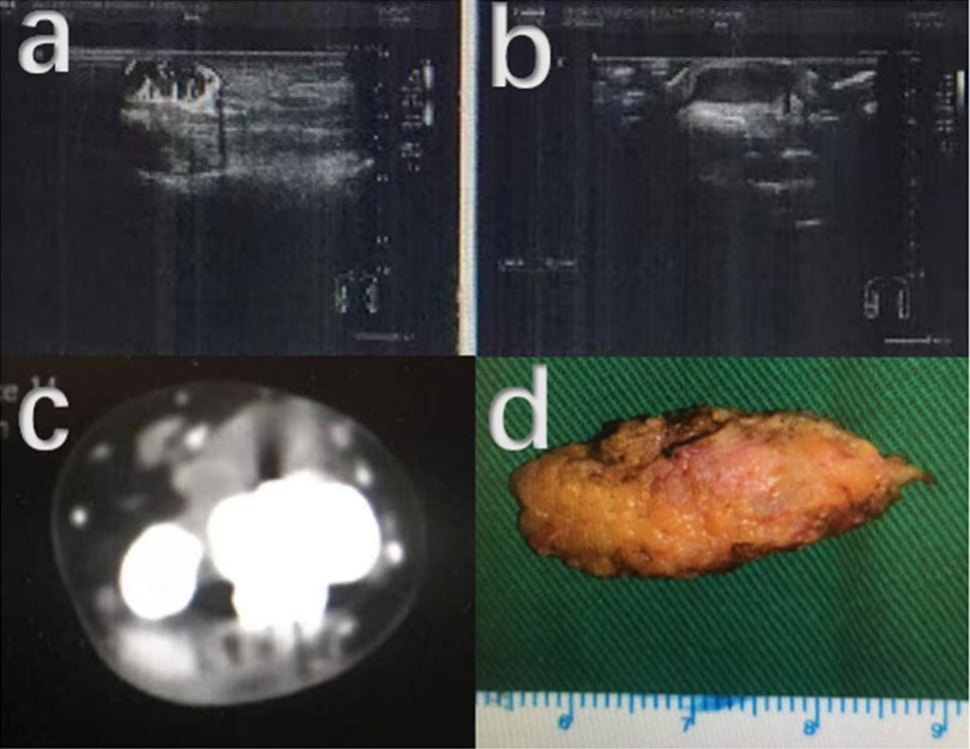
Re-recurrence of soft tissue occurred in the 22 years old female patient with GCTB located in distal radius. **a** and **b** A hypoechoic mass was detected 5 months after excision of the first recurrence. **c** CT examination before operation for the twice recurrence of soft tissue. **d** The resected soft tissue lesion

Except surgical treatment, effectiveness of adjuvant treatment with denosumab for soft tissue recurrence of GCTB was unclear. Similar to its bone recurrence counterparts, soft tissue recurrence could also develop to peripheral or intralesional mineralization, and denosumab may improve this procedure. Denosumab therapy for GCTB has been applied for years and its benefits and good therapeutic effect have been reported [16]. However, up to date, few studies have reported outcomes with denosumab application in soft tissue recurrence of GCTB. Akaike K et al. [13] and Suzuki T et al. [17] respectively reported one patient with a satisfactory outcome using denosumab. Among our 31 cases, 7 of them had a history of denosumab application, (Figs. 6, 7, 8) but the dosage and time of denosumab usage were not consistent, which made us have not conducted an effectiveness assessment of denosumab for soft tissue recurrence treatment. Among the 7 patients who received at least one dosage of denosumab, one of them had a denosumab treatment for two months before primary surgery and had two times of recurrence. Denosumab was applied in 3 patients after detection of the first recurrence, and then a reoperation was performed. One of these 3 patients underwent two times of re-recurrence, the other 2 patients had no a twice soft tissue recurrence. When a twice re-recurrence was detected, 3 other patients received denosumab treatment, one of them was still under chemotherapy for pre-existing lung metastasis, the other two had no recurrence at final follow-up. This result showed the various therapeutic effectiveness for soft tissue recurrence of GCTB in different patients. This may also be an indicator for future researches to investigate whether soft tissue recurrent patients would be benefit from adjuvant treatment with denosumab.

Location of GCTB may influence its recurrence. GCTB at distal radius showed a higher recurrence rate [1, 18]. However, whether the same is true for soft tissue recurrence remains unclear. In our cases, primary GCTBs of 8 patients with soft tissue recurrences located in radius, which accounted the most among all 31 patients (25.81%). But owing to the small number of patients in our study, we did not conduct a comparation between different locations. In another study about risk factors for local recurrence after surgical treatment of GCTB, Li D et al. [19] revealed that surgical methods, but not tumor location, pathologic fracture, age that younger than 30 years, gender and the Campanacci grade, would impact recurrence. However, we also failed to analyze primary surgical methods in our study for that some patients had received their primary surgeries in other institutions.

Campanacci grade may predict recurrence of GCTB [20]. However, our results have not validated it as a risk factor for soft tissue recurrence, and this was consistent with results of a previous multi-center cooperative research including 110 recurrent patients of GCTB [21]. The reasons may lie in that soft tissue recurrence is mainly caused by implantation during operation, while the Campanacci grade is a classification system used to reflect aggressiveness of GCTB. Thus, the Campanacci grade is more suitable for assessing recurrence in bone which may be mainly caused by residual components after non-suitable curettage or pathological fracture, rather than soft tissue recurrence which may be caused by tumor implantation during primary surgery. But the exact mechanism of soft tissue recurrence is not yet clear. Interestingly, a pathology report of one patient in our study indicated tumor embolus in a peripheral blood vessel surrounding the primary GCTB. This may indicate another underlying mechanism for soft tissue recurrence or metastasis of GCTB. However, this hypothesis needs more pathological evidence to validate.

Few researches have focused on outcomes after reoperation for recurrent GCTB, especially in soft tissue recurrence [16, 22]. Repeated recurrence of GCTB has an association with malignant transformation, and would lead to a higher incidence of metastasis to lung [21]. Early local recurrence was considered as a risk factor causing repeat recurrence in bone [21]. A median time of 13.5 months of local recurrence after GCTB resection to malignant transformation was reported by Tsukamoto, S et al. [15]. Our results showed a rate of 61.29% of patients underwent soft tissue recurrence more than once. Interval period of the first, twice and third time of recurrence gradually shortened as presented in the results section. MSTS score of these 7 patients with 3 times recurrence was slightly lower compared with the overall scoring. Among them, lung nodules were observed in 4 patients. One patient with twice recurrence died after lung metastasis. One patient with once recurrence suffered lower limb amputation at hip joint. All these indicated that soft tissue recurrence of GCTB should be given enough attention to.

This study contained some obvious disadvantages. First of all, impact of primary surgical manners of GCTB on soft tissue recurrence were not evaluated, since 15 patients received their first operations with various methods in other institutions. Experience of surgeons may not be comparable. However, no relationship between primary surgery manner and recurrence of GCTB has been reported before [21]. On the other hand, complication incidence was not compared between patients with different number of recurrences. Owing to the small number of samples and various of originate GCTB locations, we believe that it would be suitable to calculate and compare complications along with soft tissue recurrence with a larger number of patients from multi centers. Last but not the least, signal intensity in MRI was not extensively studied in this research, because we classified soft tissue recurrence mainly based on CT images. A further study concerning this aspect could be performed to reveal association between MRI presentation and prognosis of GCTB with soft tissue recurrence.

## Conclusions

Soft tissue recurrence of GCTB was less common than recurrence in bone, but it should not be ignored for the multiple recurrence possibility, and patients with soft tissue recurrence may suffer lung metastasis and death or amputation. Distal radius was the most common location of primary GCTB that develop a soft tissue recurrence. Ultrasonography is effective to detect soft tissue recurrence of GCTB. Campanacci grade and type classification, as well as our modified classification, could not predict soft tissue re-recurrence of GCTB. Therefore, a meticulous follow-up with an ultrasound examination is worth to detect the recurrent lesions early and prevent serious outcomes eventually.

## Data Availability

The datasets generated and analyzed during the current study are available from the corresponding author on reasonable request.

## Abbreviations

GCTB: Giant cell tumor of bone
DHS: Dynamic hip screw
PMMA: Polymethylmethacrylate bone cement
